# Supplementary material for: Effect of SARS-CoV-2 Infection and BNT162b2 Vaccination on the mRNA Expression of Genes Associated with Angiogenesis
Source: Int J Mol Sci. 2023 Nov 8;24(22):16094. doi: 10.3390/ijms242216094 (PMC10671623; doi:10.3390/ijms242216094)
Supplement: Supplementary file 1 [file ijms-24-16094-s001.zip › ijms-2632470-supplementary.pdf]

Supplementary Material

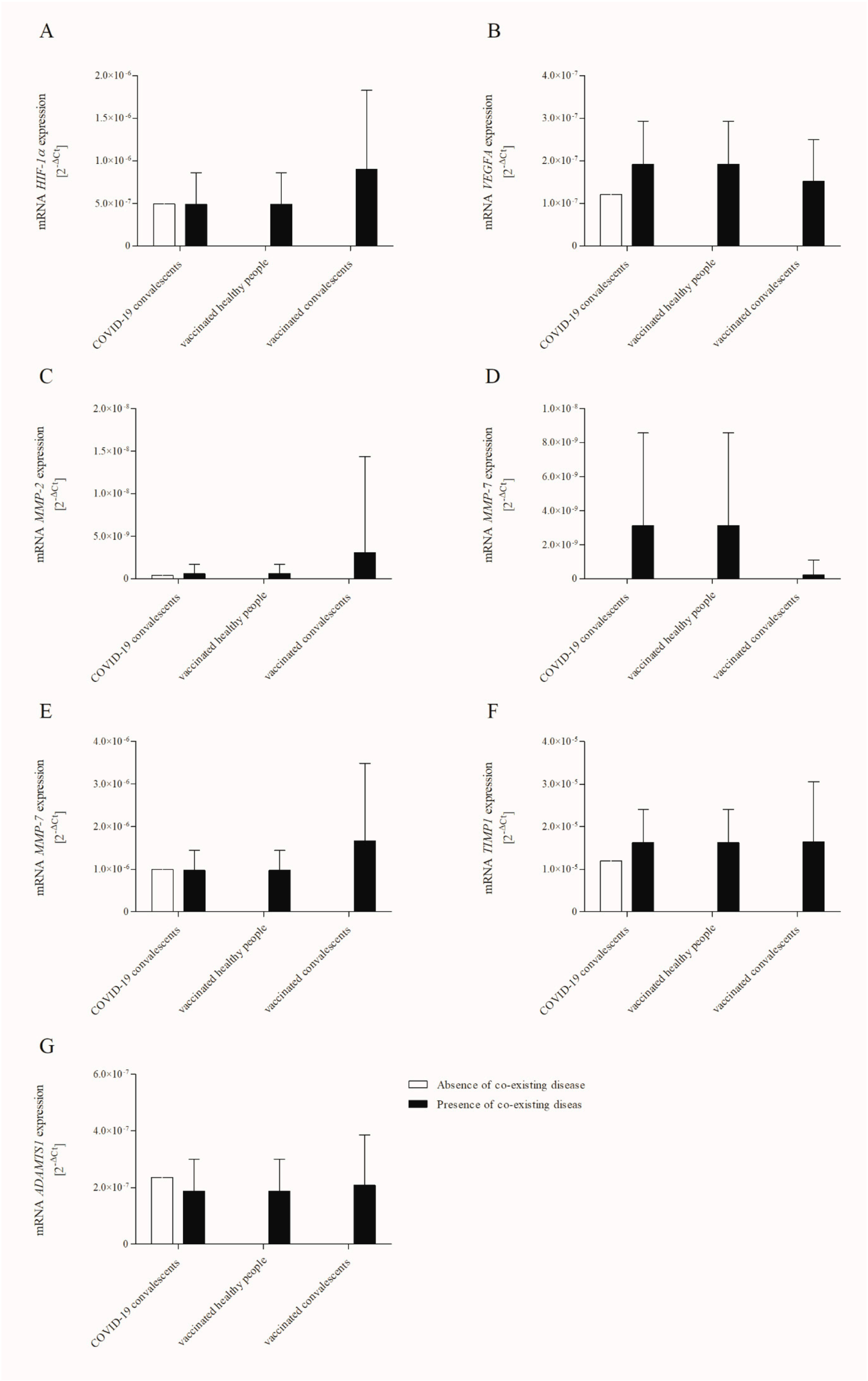

Supplementary Figure S1. Two-way ANOVA shows significant effects of absence/presence of the co-existing disease and COVID-19/BNT162b2 vaccinations on studied gene expression, including *HIF-1α* (A), *VEGFA* (B), *MMP-2* (C), *MMP-7* (D), *MMP-9* (E), *TIMP1* (F) and *ADAMTS1* (G). Relative gene expression levels were estimated using a $2^{-\Delta Ct}$ ($Ct_{\text{target gene}} - Ct_{18S}$) method. Data represent means ± SD.

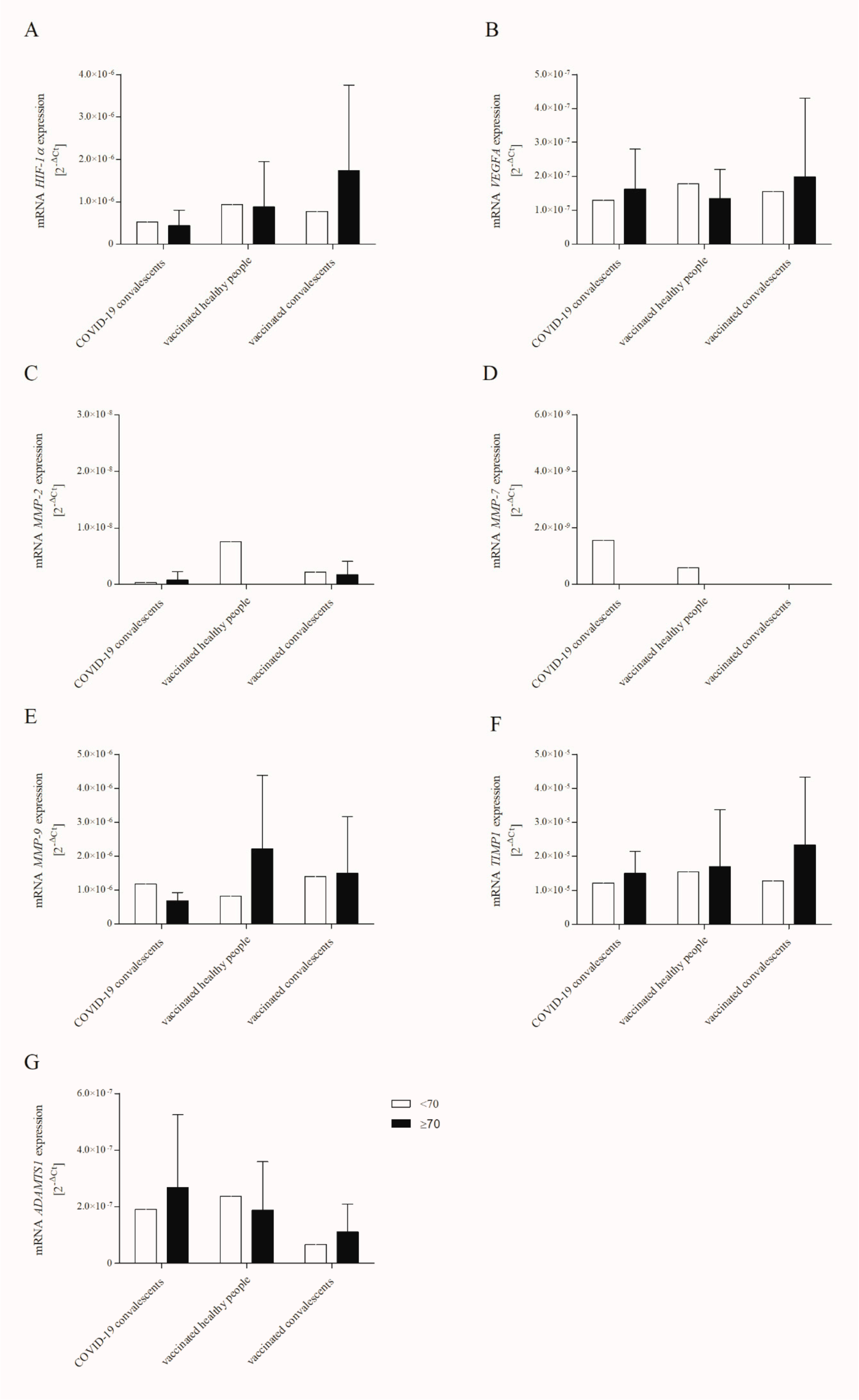

Supplementary Figure S2. Two-way ANOVA shows significant effects of age (<70 and ≥70) and COVID-19/BNT162b2 vaccinations on studied gene expression, including *HIF-1α* (A), *VEGFA* (B), *MMP-2* (C), *MMP-7* (D), *MMP-9* (E), *TIMP1* (F) and *ADAMTS1* (G). Relative gene expression levels were estimated using a $2^{-\Delta Ct}$ ($Ct_{\text{target gene}} - Ct_{18S}$) method. Data represent means ± SD.
